# Analysis of big patient mobility data for identifying medical regions, spatio-temporal characteristics and care demands of patients on the move

**DOI:** 10.1186/s12942-018-0152-x

**Published:** 2018-08-02

**Authors:** Caglar Koylu, Selman Delil, Diansheng Guo, Rahmi Nurhan Celik

**Affiliations:** 1 Department of Geographical and Sustainability Sciences, University of Iowa, Iowa City, USA; 2 Informatics Institute, Istanbul Technical University, Istanbul, Turkey; 3 Department of Geography, University of South Carolina, Columbia, USA

**Keywords:** Patient mobility, Health care, Spatial data mining, Regionalization, Flow mapping

## Abstract

**Background:**

Patient mobility can be defined as a patient’s movement or utilization of a health care service located in a place or region other than the patient’s place of residence. Mobility provides freedom to patients to obtain health care from providers across regions and even countries. It is essential to monitor patient choices in order to maintain the quality standards and responsiveness of the health system, otherwise, the health system may suffer from geographic disparities in the accessibility to quality and responsive health care. In this article, we study patient mobility in a national health care system to identify medical regions, spatio-temporal and service characteristics of health care utilization, and demands for patient mobility.

**Methods:**

We conducted a systematic analysis of province-to-province patient mobility in Turkey from December 2009 to December 2013, which was derived from 1.2 billion health service records. We first used a flow-based regionalization method to discover functional medical regions from the patient mobility network. We compare the results of data-driven regions to designated regions of the government in order to identify the areas of mismatch between planned regional service delivery and the observed utilization in the form of patient flows. Second, we used feature selection, and multivariate flow clustering to identify spatio-temporal characteristics and health care needs of patients on the move.

**Results:**

Medical regions we derived by analyzing the patient mobility data showed strong overlap with the designated regions of the Ministry of Health. We also identified a number of regions that the regional service utilization did not match the planned service delivery. Overall, our spatio-temporal and multivariate analysis of regional and long-distance patient flows revealed strong relationship with socio-demographic and cultural structure of the society and migration patterns. Also, patient flows exhibited seasonal patterns, and yearly trends which correlate with implemented policies throughout the period. We found that policies resulted in different outcomes across the country. We also identified characteristics of long-distance flows which could help inform policy-making by assessing the needs of patients in terms of medical specialization, service level and type.

**Conclusions:**

Our approach helped identify (1) the mismatch between regional policy and practice in health care utilization (2) spatial, temporal, health service level characteristics and medical specialties that patients seek out by traveling longer distances. Our findings can help identify the imbalance between supply and demand, changes in mobility behaviors, and inform policy-making with insights.

## Background

Designing a well-functioning health care system that is accessible, high quality and affordable is challenging as it requires balancing supply and demand for quantity, quality and variety of specialized medical care. Patient mobility can be defined as a patient’s movement or utilization of a health care service located in a place or region other than the patient’s place of residence. Mobility provides patients a wider choice of providers and increases the competition in health care market [1] and the efficiency of the health system [2]. Patients who are in search for immediate, affordable, and unusual treatments travel long distances, and often go beyond the conventional territorial boundaries [3, 4]. Patient mobility has been used as a policy to provide accessible and equitable care across the world. For example, the European Union have implemented policies to support free movement of patients across the countries in the EU [5].

Patient mobility research has typically focused on transnational patient movements across countries and continents within the context of medical tourism [6, 7]. A growing body of research is increasingly recognizing the importance of patient mobility to address the issues such as high cost of long waiting list at home, new technology, and skills in destination areas and countries [8, 9]. Furthermore, reduced transport costs resulted in increased medical tourism that go beyond the borders of countries and even continents. Health care providers and planners have implemented policies and various techniques to create and implement medical regions in order to efficiently allocate resources and services [10–12]. Each medical region includes a hub which provides both quantity and variety of care for patients for the surrounding areas. The health system can function more efficiently if it is organized into functional regions where the size and characteristics of the population, the quantity of providers and type of health care needs are known. In this article, we study interregional patient mobility in Turkish Health System.

Glinos et al. [3] identify availability, affordability, familiarity, and perceived quality as the four major motivations for patients that seek health care elsewhere. Availability refers to both timely access to services (i.e., short waiting list) and types of specialized care services within the residents’ area. Affordability allows patients to choose the most economical care. Familiarity represents the cultural closeness or the availability of family and social ties in destination location. Lastly, perceived quality refers to the patient’s perception of the quality and safety of services, technology and methods used at the destination location. Additionally, indicators of geographic accessibility to health care [13, 14], and financial accessibility and acceptability [15] have been considered to impact the choice of patients [4].

Although mobility increases competition in the market, it is essential to monitor the patterns and shifts in patient choices in order to identify the gap between what patients need and what the health system offers in terms of quality care and services [6, 16, 17]. Otherwise, the health system may suffer from geographic disparities in the access to quality and responsive health care. Furthermore, monitoring changes in mobility behaviors can help identify the imbalance between supply and demand and inform policy-making with insights.

Analysis of mobility data is challenging due to the large volume and number of flow variables (e.g., hundreds of variables including service levels, types and medical specialization); and the complexity of patterns in multiple spaces (e.g., geographic space, network space, multivariate space) and time, and at multiple scales (e.g., national patterns, regional patterns, local patterns). Analysis of mobility networks can be grouped into four major themes: (1) using network measures [18] to identify locational characteristics such as prominent nodes and popular destinations; (2) using community detection methods to identify functional regions such as medical regions [11], habitat territories [19], or migration regions [20, 21] where there are more flows within a spatial community (a set of locations) than the rest of the network; (3) using flow clustering, edge bundling and visualization [22, 23] to identify common characteristics of flows (4) using global statistical and network models such as power-law, and gravity to describe the governing laws behind the network construction, evolution, and distributional characteristics [24, 25].

In this article, we present a systematic approach to analyzing large and high dimensional (i.e., large number of variables) patient mobility data in order to evaluate the effectiveness of regional health care utilization and identify the characteristics and demands for long-distance patient mobility. To demonstrate, we analyze patient mobility in the Turkish National Health System across a 4-year period from December 2009 to December 2013. Mobility data is derived from 1.2 billion monthly aggregated health service records, which include locations of patients and health service facility as well as multivariate information on service provider, type of institution, and level and specialization of medical service received. We construct a province-to-province patient mobility network, one for each month, where nodes represent locations of patient residences and health service facilities, and edges represent monthly-aggregated movement of patients from the province of their residences (origin) to the province of health facilities (destination). We first derive functional regions of health service delivery by applying a flow-based regionalization approach [26] to the province-to-province mobility network. We compare the data-driven regions to the designated regions from the Turkish Ministry of Health in order to capture the mismatch between the implemented regional policy and observed choices of patients. Functional regions derived from patient mobility data can help evaluate the effectiveness of the regional health system. Second, we identify the spatio-temporal characteristics and health care demands of moving patients by performing feature selection and multivariate clustering on a large set of patient flow variables which include time (e.g., month, season, and year), type, level and specialization of medical services received by patients. Such information can help identify the imbalance between supply and demand, changes in mobility behaviors, and inform policy-making with insights.

## Methods

### Context and data

In this section, we introduce the details of the Turkish Healthcare system and the patient mobility data. Health providers in Turkey are organized into three levels (Table 1): (1) primary health centers staffed by family physicians (2) public and private health facilities and hospitals, and (3) training and research hospitals and independent university hospitals.

**Table 1:** Health care service levels in Turkey

	Primary	Secondary	Tertiary
Facility	Community health centers, family health centers	Public and private health facility and hospitals	Training and research, and university hospitals
Staff	Family physicians	Specialist physicians	Specialist physicians and medical residents

#### Turkish health system

**Fig. 1:**
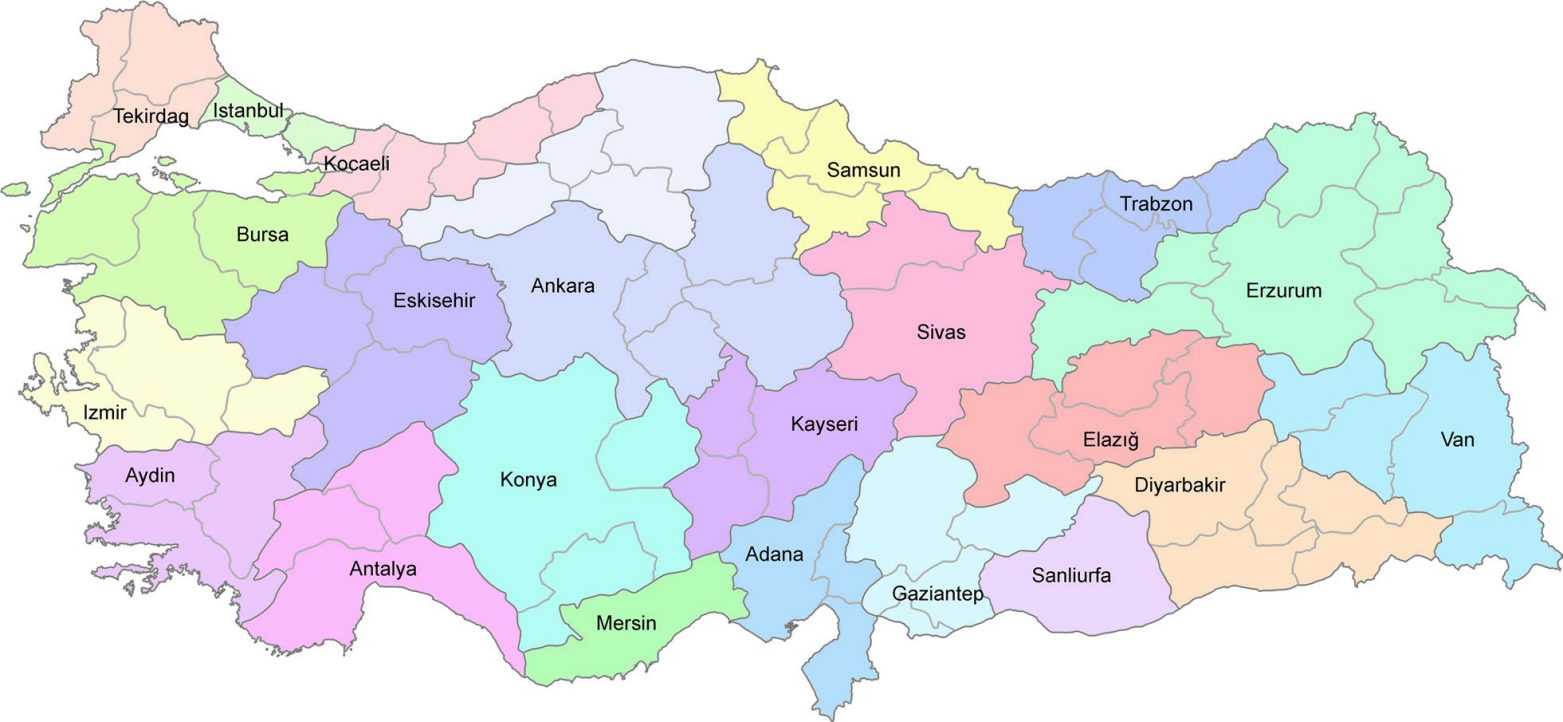
Ministry of Health (MoH) designated health care regions and hubs assigned to each region. Hubs are illustrated by the province labels

Turkey’s health system has radically changed with the Turkish Health Transformation Program (THTP) in 2003, which aimed to improve governance, efficiency and quality in the health care sector, with significant investments and the establishment of a family-physician system [27]. Employees in both public and private sectors were combined under the newly created Social Security Institution (SSI) and almost the entire population was covered by social insurance with universal health coverage. After many legislative changes, the SSI has become the sole buyer of health care services and the Ministry of Health (MoH) has become the main health care service provider in the country. With the implementation of THTP, citizens were given the freedom to choose where they are treated, whether in a private or a public institution without referral requirement or out-of-network coverage. In 2011, MoH released a new region-centered planning policy and family physician system, which combined eighty one provinces into twenty nine health regions. Figure 1 shows the regions and the health care hub designated for each region. According to the new policy, each patient was assigned to a family physician, and patients in each region were advised to seek specialized medical care from their regional hub before considering hubs in other regions. However, since there are no referral requirements, and citizens are free to choose health services with their universal health insurance, it is not clear whether the MoH regions and hub structure function effectively in practice. One of the objectives of this paper is therefore to identify the functional regions directly from the patient mobility data, and compare them to the designated regions by MoH to assess the effectiveness of the implemented policy.

#### Patient mobility data

After the health care reform, health care utilization has increased dramatically and patient mobility has continued to rise in Turkey. Main drivers of patient mobility are the search for better treatment or treatment affordability, and the availability of specialized health care services [28]. After the Social Security Institution (SSI) became the single buyer for health care services, different databases of providers and related insurance coverage were merged into a single database. The SSI health insurance covers 90% of the entire population in Turkey, a total of about 70 million. On average 400 million admissions are provisioned annually by health service providers. These records include the number of patients admitted to health care units on a monthly basis, and are verified and monitored by SSI using a single-center database. Both patients’ residence location and health care unit or facility location are verified by the Turkish address-based population registration system (TABPRS). Because the data include all health service records, it potentially involve multiple trips from the same individual across 4 years. Because the data is aggregated by month and province and district to protect the privacy of patients, it is impossible to identify the individual trajectory of patients.

We acquired a 4-year dataset of patient mobility between December 2009 and December 2013, which includes more than 1.2 billion health service records. Each record has specific mobility information, including the visit time (year and month), the residence location of the patient at the district level, location of the health service facility at the province level, and service provider, type of institution, and category of medical service received (Table 2). Location information was not available for 11% of patients, who are either non-citizens from cross-border medical tourism or those that do not have a citizenship ID in the system. We excluded these records which do not have home residence information.

**Table 2:** Patient admission records and attributes

Time period	December 2009 to December 2013 (48 months)
Origin	Patient’s residence location at the district level
Destination	Health care unit location at the province level
Flow attributes	Institution: 12 types indicate the service provider and institution (e.g., public, private, university) Service level: (primary, secondary, and tertiary care) Service category: 120 medical specialties

The patient mobility data naturally form a bipartite spatial network where there are two types of nodes: patient residences and health facilities. An edge (flow) in this network represents a patient’s mobility from her/his residence (origin) to a health facility (destination). Provinces are divided into counties and counties are divided into districts in Turkey’s hierarchy of administrative units. While the residence of a patient is provided at district level, the health facility that the patient was admitted is provided at province level. In order to match the spatial resolutions of origins and destinations, we generated a weighted and directed network of province-to-province patient flows in which a node represents a province, whereas an edge represents monthly aggregated mobility of patients from the province of their residence to the province of the health facility. In addition to the total volume of each flow, we also aggregated the multivariate characteristics of each flow such as the type of service provider and the medical specialty by province-to-province pairs, with counts for each category.

### Methods

#### Definitions

A longitudinal set of origin–destination (O–D) mobility network (graph) can be formulated by a sequence of non-overlapping time windows, each of which represents a snapshot of flows within that time window. Starting at time $t_{min}$ and ending at $t_{max}$, we use the notation $M_{t}^{w} \left( {t_{min} , t_{max} } \right)$ to describe a time-ordered sequence of mobility graphs, $M_{tmin}^{w} , M_{tmin\, + \,w}^{w} , \ldots , M_{t}^{w} t_{max }$, where w denotes the size of each time window in some time unit (i.e., hours, days, weeks, months). Each O–D graph $M_{t}^{w}$ within the sequence is defined by a set of locations $P = \left\{ { p_{1} , \ldots , p_{n} } \right\}$ and a set of flows (edges) $F = \left\{ {f_{ij}^{s} } \right\}$, where $i \in P,\;j \in P,\;i \ne j$, $t\, \le \,s\, \le \,t\, + \,w$, and $f_{ij}^{s}$ represents the flow from i to j within the time window s. In an O–D graph $M_{t}^{w}$, each location has fixed geographic coordinates, however, their existence depend on the existence of flows to and from that location within the given time window. By changing the size of the time window, one can obtain different levels of granularity in temporal scales. Selecting an appropriate size for time window is an application specific problem, as windows with different sizes may help capture different meaningful behavior, however, the maximum temporal resolution is preferred to be able to capture the finest differences between each graph [29]. On one end, the maximum temporal resolution results in each interval corresponding to the smallest time unit, or to the time between any two consecutive modification of the graph [30]. On the other hand, the minimum temporal resolution would correspond to a single graph which aggregates all interactions over time. In this paper, we first use the minimum temporal resolution, and consider the full dataset to identify consistent functional regions and multivariate flow patterns. Secondly, we use the maximum temporal resolution of monthly O–D graphs to identify spatiotemporal flow patterns that characterize the patient demands.

#### Locational measures of mobility

In order to identify the places of attraction and depletion, we compute locational measures of net-flow ratio and gross flow for the whole 4-year period, and the years 2010–2011 and 2012–2013.

$$\begin{aligned} & Netflow_{i} = Inflow_{i} - Outflow_{i} \\ & Gross\,flow_{i} = Inflow_{i} + Outflow_{i} \\ & Netflow\;Ratio_{i} = \frac{{Netflow_{i} }}{{Grossflow_{i} }} \\ \end{aligned}$$

Net-flow ratio for a location i is calculated by dividing the net-flow (i.e., total patients in minus total patients out) by the total flow of patients in and out of a province.

#### Hierarchical regionalization of flows

We first derive functional regions of patient flows using a flow-based hierarchical regionalization, or in other words, spatially-constrained graph partitioning approach. A hierarchy of regions allows capturing mobility patterns at different scales such as the national scale, regional and provincial scale. For example, when looking at 4-region partition one can better understand patterns at national scale, while patient mobility at 23 regions helps understand patterns at regional scale and allow comparison with the designated regions of Ministry of Health. We compare the results of data-driven regions to designated regions of the government in order to identify the areas of mismatch between planned regional hub structure and the observed structure of the patient flows. Different from community detection in non-spatial networks, the objective of a spatially or contiguity constrained graph partitioning is to identify functional regions by grouping strongly connected and spatially adjacent nodes into clusters. We adopt the flow-based hierarchical regionalization approach [26] which consists of the following steps. First, we convert raw flow matrix into a modularity matrix in order to remove the effect of size differences among the locations (nodes) in the O–D network. Each edge in the modularity matrix represents the modularity between a pair of provinces in two directions, which is derived by the difference between the actual flow and the expected volume of flow for each pair of locations. A variety of statistical measures can be used to calculate the expected volume of flows, we employ the following formula which is based on an adjusted flow volume:

$${\text{EF}}\,\left({\text{O,D}}\right)={\text{F}}_{\text{O}}\,{\text{F}}_{{\rm D}}\,{\text{f}}\left({{\text{O,D}}}\right)/\left({{\text{F}}_{\text{S}}^{2} - \mathop{\sum}\limits_{i = 0}^{n} F_{i}^{2} } \right)$$

where F_O_ is the number of flows between area O and its connections, F_D_ is the number of flows between area D and its connections, f(O, D) is the number of flows between area O and area D, F_S_ is the number of flows between all areas (provinces), and $\mathop \sum \limits_{i = 0}^{n} F_{i}^{2}$ is used to remove within-area expectations. Finally, modularity of a link O–D is calculated as:

$${\text{MOD}}\left( {{\text{O}},\;{\text{D}}} \right)\; = \;{\text{AF}}\, - \,{\text{EF}}$$

where AF is actual number of flows, and EF is expected number of flows on the link O–D. Using this formula, we transform the O–D network of raw counts of patient flows into an O–D modularity graph, in which the weight of a link represents the modularity between two locations. If modularity value is positive the link is considered to be above expectation, if the value is negative the link is below expectation.

Second, we perform a full-order average linkage clustering (ALK) in order to construct a set of spatially contiguous regions. ALK is a clustering method that builds a hierarchy of spatially contiguous clusters by iteratively merging the most connected clusters. The method first produces a spatially contiguous tree, where each edge connects two geographic neighbors and the entire tree is consistent with the cluster hierarchy. Then each region in the spatially contiguous tree is partitioned into two regions based on an objective function which maximizes within-region modularity for each region (community). The modularity is calculated by the sum of flow-expectation difference for each pair of units inside a region and for all regions. We used the software tools developed in Java for regionalization and flow mapping whose further details can be found in [26].

#### Feature selection

Visual analytics allows users to explore hidden patterns in the data by integrating computational methods with multiple perspectives provided by dynamically-linked visualization of patterns in geographic space, attribute space and time. We used visual analytics to capture the complex patterns of patient flows, where they are from, where they are going to, what characteristics they have, and what regions they form. The visual analytics tools used in this study can also be used in any flow analysis such as migration, commuting, traffic flows or waste flows.

We first identify the spatio-temporal characteristics and health care needs of moving patients by performing feature selection using a visual analytics environment. There are four main groups of patient flow variables: the visit time (i.e., month, season, year), service provider (i.e., public, private, university); service level (i.e., primary, secondary, tertiary), and service category which includes 120 medical specializations such as Cardiology, Urology, and Gynecology. Large number of variables pose a significant challenge for flow pattern analysis, and we initially do not know which variables are relevant and can be useful to characterize or predict the mobility behaviors. In order to provide a comprehensive analysis framework that take into account the interaction between various flow variables, we use a feature selection [31] and clustering methodology [26].

**Fig. 2:**
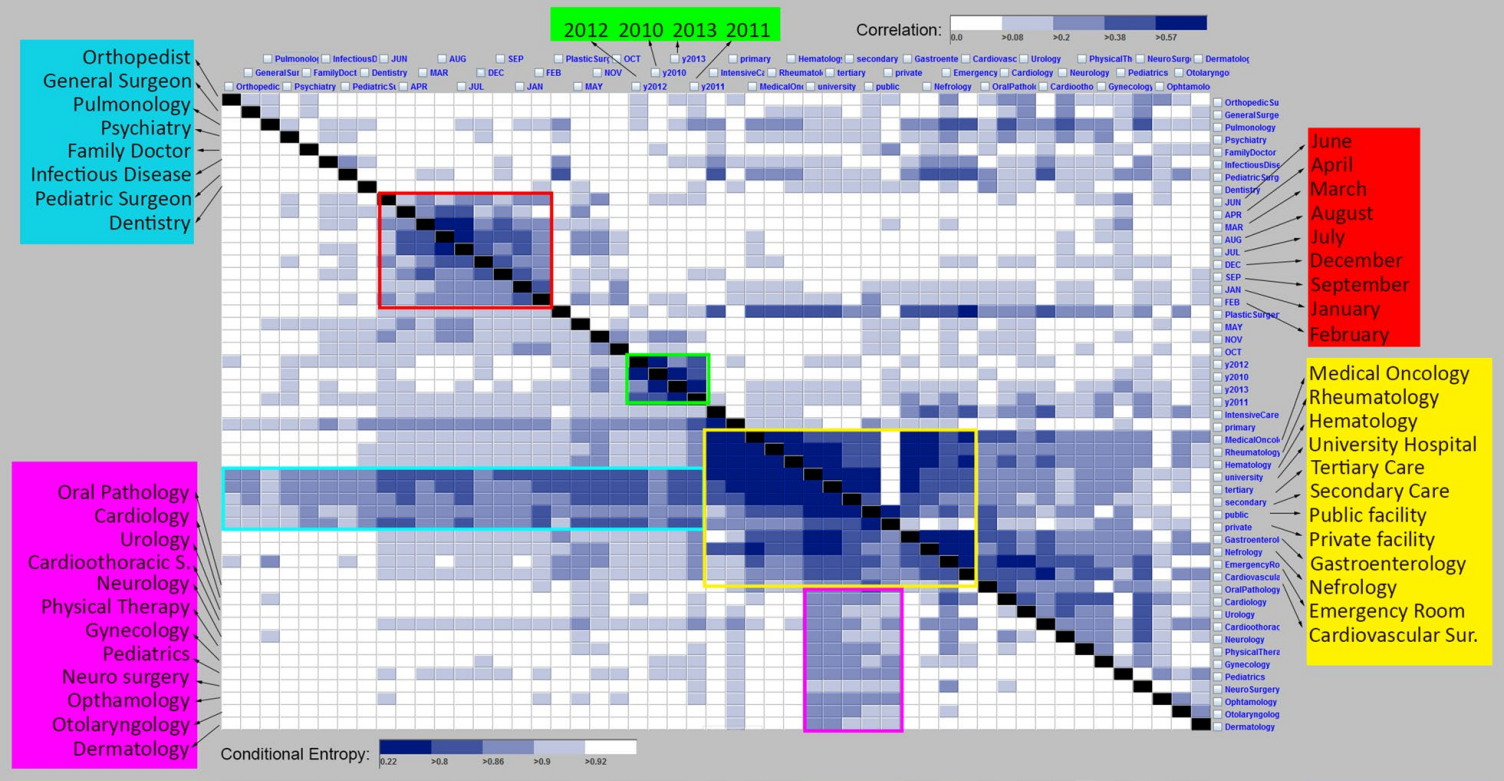
Selection of flow variables using conditional entropy (bottom left) and correlation (top right)

For selecting interesting group of variables, we use degree of correlation and clustering between every pair of attributes to determine bivariate attribute pairs that contain interesting patterns. While correlation quantifies the linear relationship between a pair of variables, maximum conditional entropy (MCE) provides a measure of “goodness of clustering” between two variables. We first construct a feature similarity matrix that illustrate bi-variate similarity between every pair of variables both in terms of correlation and clustering, we then reorder this matrix in a way that higher values of entropy are next to each other and closer to the diagonal section of the matrix (Fig. 2). In the feature matrix, each cell with a color illustrates a measure value between two attribute pairs. The pairwise conditional entropy values of all dimensions are shown in the bottom left part of the matrix in which lower entropy values (darker colors) represent more interesting subspaces. On the other hand, the pairwise correlation values of all dimensions are shown in the top right of the feature selection matrix, and higher correlation values (darker colors) represent strong relationship between variables. In both sides the darker colors illustrate more interesting relationships between each pair of variables. The ordering of the variables in the matrix is derived from the entropy matrix using a minimum spanning tree (MST) in order to group the cells with higher interestingness to reveal potentially interesting variable subspaces [31].

We selected five interesting subspaces from the feature matrix which are highlighted in red, green, purple, cyan, orange, and yellow in Fig. 2 in order to guide our analysis of flow patterns. Red subspace consists of monthly variation of flows (i.e., January to December); green subspace consists of yearly aggregation of flows from 2010 to 2013; yellow subspace consists of a combination of flow variables that include type of institution and medical service; cyan subspace consists of variables of mostly monthly aggregations, some types of institution, and a few types of medical service; and purple subspace consists of types of institution, and certain types of medical service. In this paper, we provide a further analysis of flow patterns using the top four subspaces: red, green, and yellow in Fig. 2 which were identified as interesting by both conditional entropy and correlation measures. These subspaces illustrate the demands of patients that move long distances between regions.

#### Multivariate clustering

**Fig. 3:**
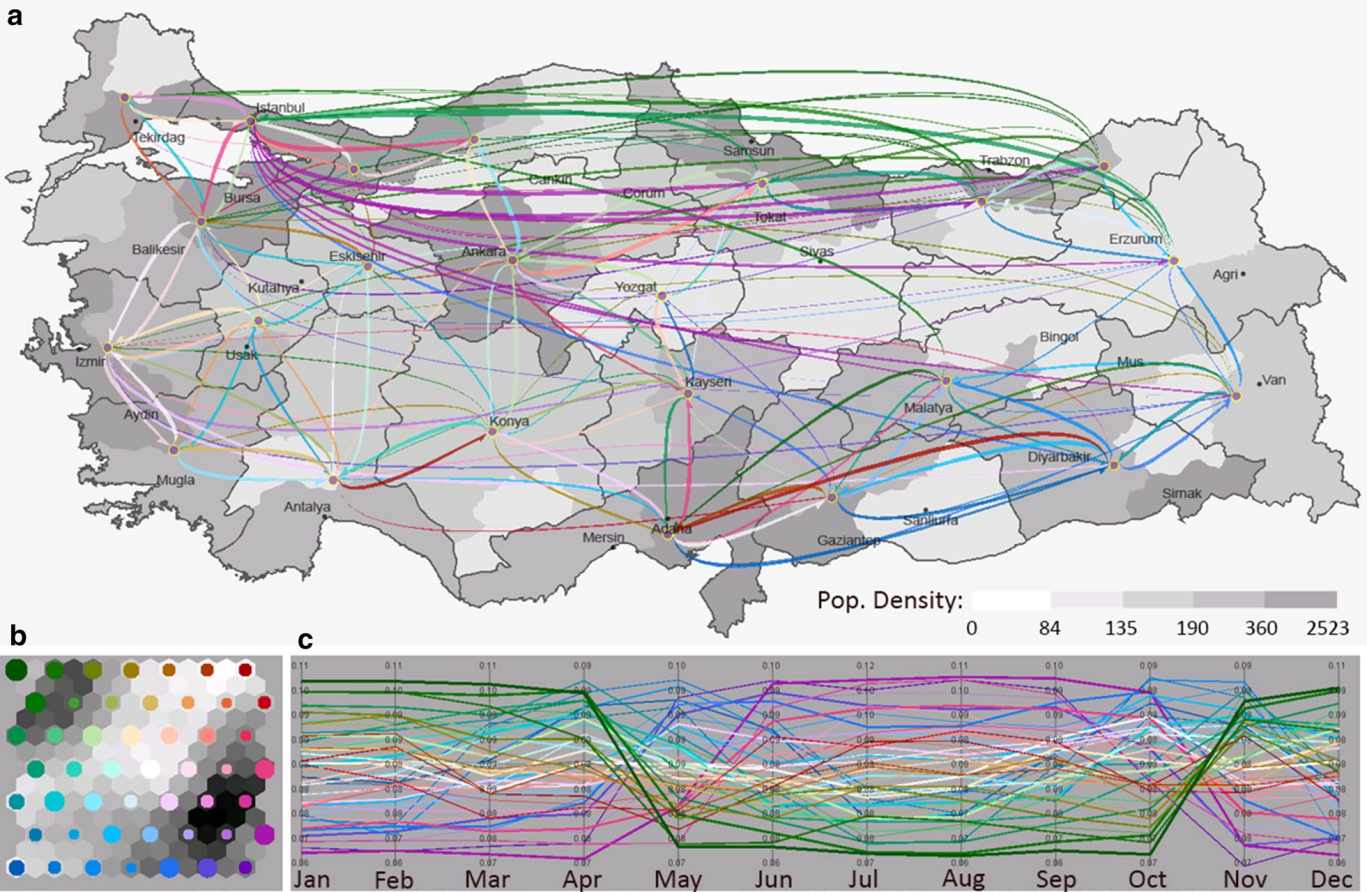
Monthly patterns of patient flows **a** flow map, **b** self-organizing map, **c** parallel coordinate plot

We use an integrated visual analytics framework [26] that combines flow mapping with multivariate clustering and visualization (Fig. 3). The integrated framework uses the pre-selected subspaces of variables in the first task (i.e., collection of variables included within red, green, cyan, yellow and purple rectangles), and help identify the multivariate relationships between the variables of each subspace, and their spatial flow patterns. A self-organizing map (Fig. 3a) is used to order the multivariate clusters of flows in a two-dimensional layout in which nearby clusters are similar in terms of their flow attributes. Each SOM node (circle) illustrates a cluster of flows, and the size of each circle represents the number of flows the cluster contains. The hexagons that are drawn under each circle represent the multivariate dissimilarity between neighboring nodes, where darker tones illustrate greater dissimilarity. A 2D color scheme is used to assign each SOM cluster a unique color and similar clusters have similar colors. The colors created by the SOM are then passed onto a flow map that illustrates spatial patterns of flows (Fig. 3a) and a parallel coordinate plot (Fig. 3c) that reveals the meaning of each multivariate cluster. Figure 3 illustrates the multivariate clusters defined by the red subspace in Fig. 2 which corresponds to the monthly patterns of patient mobility. The flows are symbolized based on the proportion of the total volume of movement for a specific month to the total volume of flows for the whole time period. In Fig. 3, green clusters of patients who are residents of eastern regions and seek health services in large cities of Istanbul, Ankara, Izmir and Adana during the months from November to April. The purple clusters of patients are residents of large cities that seek care in the north, north-eastern and south-eastern regions. In addition, blue clusters of patient flows in Fig. 3 characterizes the short-distance mobility of patients mostly in the South-East which peaks during spring and autumn months.

## Results

We introduce the results of our analysis in three sections. The first section reports on the temporal and service level patterns. The second section introduces the functional regions derived from the patient mobility data, and compares the regionalization result to that of designated regions by Ministry of Health (MoH). The third section introduces the spatio-temporal and multivariate flow patterns that help characterize the demands of patients.

### Temporal and service level patterns

**Fig. 4:**
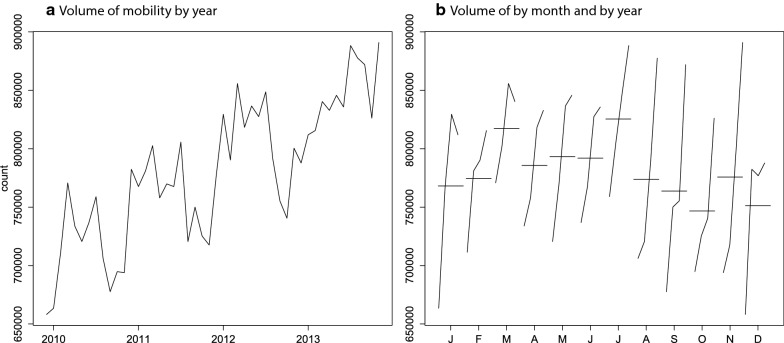
Total patient mobility **a** by year, **b** by month of the year

In order to answer the question how has mobility changed over time?, we provide a summary of the total hospital admission records and mobility by year in Table 3, and we illustrate change in mobility by year and month in Fig. 4. Although health service use and mobility increased over time, yearly mobility ratio declined from December 2009 to December 2013. The volume of patient mobility between provinces follows an increasing trend with seasonal fluctuations and high volumes in summer months for the study period (Fig. 4a). Figure 4b illustrates monthly total mobility volumes across the 4-year period. The x-axis represents months, and the vertical lines on each month represents the yearly variation in monthly volume of mobility across the 4-year period. For example, for January and March, total volume of mobility increased steadily, however, it declined in year 2013 (see the declining lines in Fig. 4b). Horizontal lines are used to represent the average of the 4-year period for each month. Spring and summer months exhibit higher volume of mobility than autumn and winter. March and July are the peak months, whereas October and December exhibit the lowest patient mobility across the 4-year period. The range of values during each month of the 4-year period was between 100 thousand and 200 thousand. Although summer months resulted in more patient mobility, patient mobility in winter months increased greater than the summer months over the 4-year period.

**Table 3:** Health service records and mobility ratio by year

Time interval	# admissions	# mobility	Mobility ratio (%)
December 2009–November 2010	251,630,100	32,843,706	13.05
December 2010–November 2011	292,626,833	36,407,051	12.44
December 2011–November 2012	355,843,020	41,755,845	11.73
December 2012–November 2013	372,586,211	43,772,750	11.75

**Fig. 5:**
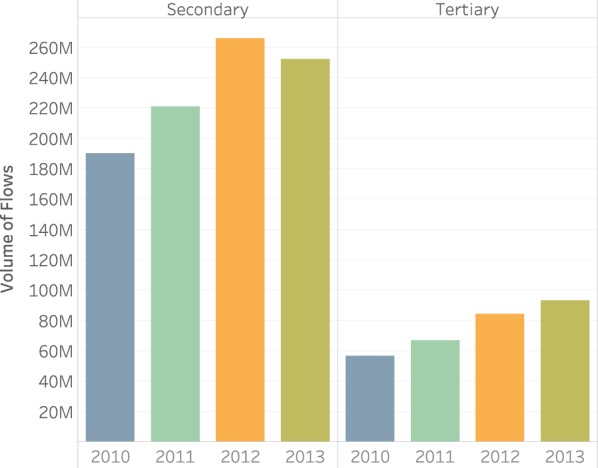
Patient mobility between provinces by service level and year

Figure 5 illustrates the mobility by health care service levels such as primary, secondary and tertiary care, and by years. Secondary care includes most of patient mobility between provinces with an average of 232 million patient flows per year, and patient mobility demanding secondary care increased over years with a decline in 2013. Patients seeking tertiary care in other provinces steadily increased over time, however, the total number of patients for the 4 year average was around 75 million. Figure 5 does not display primary care patient flows between provinces since the numbers were very marginal as compared to secondary and tertiary care. In the years of 2010 and 2011, there was an average number of 713 thousand patients that received primary health care in a province other than the province of their residence. Strikingly, this number went down to only 86 and 128 for years 2012 and 2013 respectively. We can attribute this change to the policy change in 2011 that assigned each patient to a family physician.

### Spatial patterns

#### Patterns of attraction and depletion

**Fig. 6:**
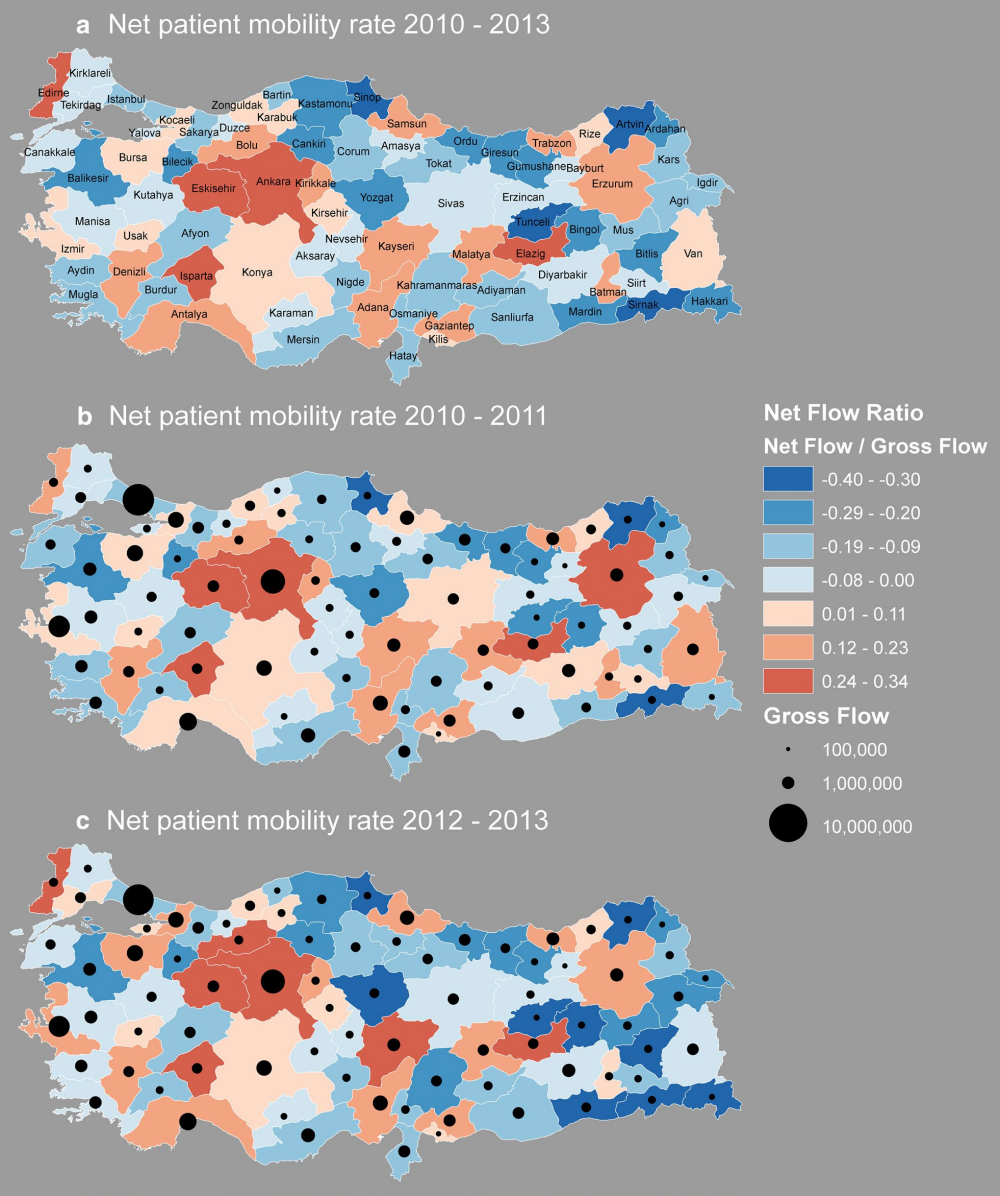
**a** Net patient flow ratio for the 4-year period, **b** net flow ratio and gross flow in 2010–2011, **c** net flow ratio and gross flow in 2012–2013

In order to reveal provinces that attract and push patients for health care service, we compute a series of patient flow ratio measures. Flow ratio is calculated by dividing the net flow (inflow–outflow) by the gross flow (the summation of total inflow and outflow) of patients per province. Figure 6 illustrates the net flow ratio for each province including all 4-year period (Fig. 6a), and net flow ratio and gross volume of flows per province for the time periods 2010–2011 (Fig. 6b) and 2012–2013 (Fig. 6c). These time periods correspond to the before and aftermath period of the policy change on the establishment of family physicians implemented in 2011 and put in place in 2012. Overall, orange colors highlight the provinces of Ankara and Eskisehir in central Anatolia, Isparta in south west; and Elazig in the east and Edirne in north west as major places of attraction for patients. It is remarkable to see that even though it has the largest number and variety of medical services, Istanbul has a negative net flow ratio, which highlight the excessive number of patients who reside in Istanbul but receive health care elsewhere. On the other hand, Fig. 6 also highlights many small provinces such as Sinop, Kars and Erzincan where patients travel to other provinces to receive health care. These provinces cluster in the south east, north and north east. In addition to general patterns of health care utilization by province, Fig. 6b, c provide a comparison of the net flow ratio as well as gross flows in the first and second half of the dataset in order to reveal changes after the family physician policy implemented in 2011. A remarkable difference between the two time periods is that the values were stretched towards positive and negative outliers, and as a result we observe darker blue and darker red provinces that indicate increased attraction and depletion. It is clear from the two figures that patient mobility out of the eastern provinces increased in the period after the policy change, which is highlighted by large negative net flow ratio (dark blue colors). We attribute this change to the establishment of the family physician service which increased the number of referrals to specialist that are located elsewhere. On the other hand, central Anatolia including Ankara, Eskisehir and Bolu became central places of attraction for patients across the nation. We also observe an increase in net flow ratio in provinces such as Kayseri, Edirne, Bursa and Kocaeli.

#### Functional regions

**Fig. 7:**
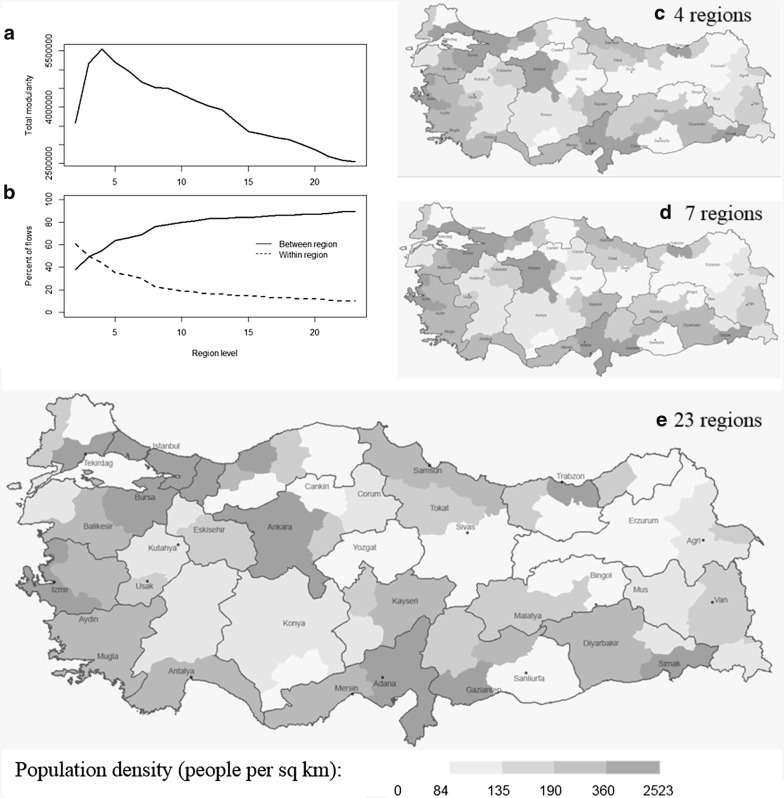
**a** Within-region modularity, **b** percent flows within and between regions. Hierarchical regions of patient mobility at **c** four regions, **d** seven regions, and **e** twenty three regions

We employed the Average Linkage Clustering (ALK) to derive a hierarchy of regions using the minimum temporal resolution, i.e., the mobility graph for the whole period $M_{t}^{w} \left( {t_{min} , t_{max} } \right)$. We computed the total within-region modularity of the hierarchical partitions to evaluate the partitioning result at different levels up to twenty three regions which we used as a baseline to compare with the designated regions by MoH. While each partition level highlights patterns at different scales, the partition with four regions maximizes the total within-region modularity (Fig. 7a), therefore, suggests a stable partitioning of the patient mobility network for the discovery of community structures. As the number of regions increases, the percentage of flows between regions naturally increases. While 50% of flows were between regions for the four-region partition, 89% of flows were between regions for the twenty three-region partition (Fig. 7b). Among the four regions of Anatolia (i.e., the main land of Turkey), the northern region is formed by the provinces that are tightly connected to Istanbul (Fig. 7c). The inland and western regions are very consistent with the hinterlands of the two big cities Ankara and Izmir. The south-eastern region is formed by merging of the hinterland of Antalya and Diyarbakir. These two provinces were designated as major health care hubs by Ministry of Health (MoH) for the surrounding provinces in their allocated regions. Seven-region partition results from further partitioning of the north, west, and south-east regions observed in the four-region partition (Fig. 7d). MoH designated health care regions, and assigned a hub for each region. These hubs were either already functioning as hubs or MoH planned these hubs as future hubs by investing in the health infrastructure to serve the surrounding provinces that lack capacities and certain specialized services. The original designation by MoH consists of twenty nine regions which separates the metropolitan areas of Istanbul to six sub regions, Ankara to two regions. In order to compare the designated regions of MoH with the regions derived from flows of patients between provinces, we used Istanbul and Ankara in their own regions without splitting these provinces into sub-regions. As a result, the number of designated regions went from twenty nine down to twenty three. Figure 7e illustrates the twenty three-region partition in order to compare with the designated regions defined by MoH.

**Fig. 8:**
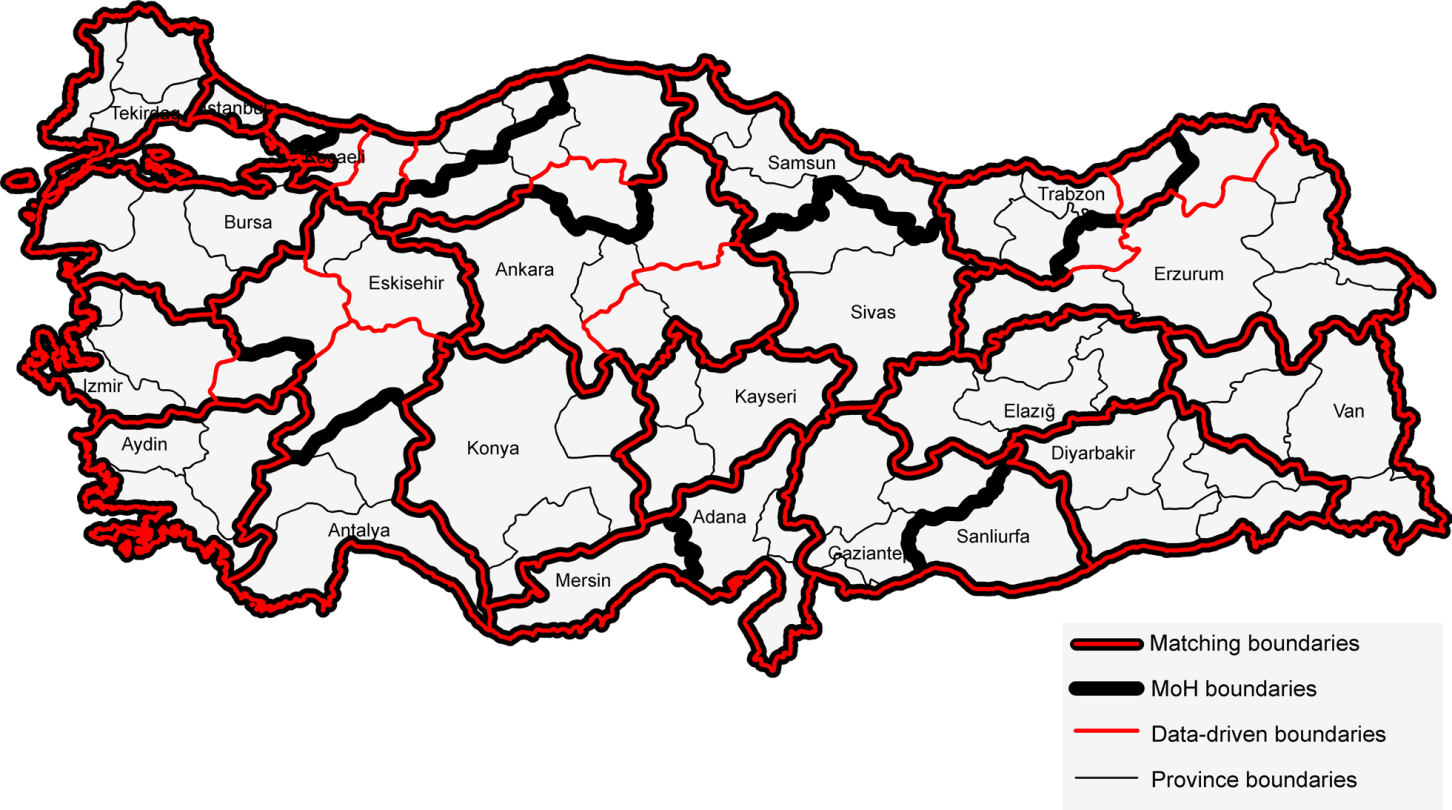
Comparison of Ministry of Health’s designated regions with the data-driven regions

Figure 8 illustrates the comparison of Ministry of Health (MoH) designated regions and twenty three regions derived from patient mobility. Boundaries of designated regions by MoH are shown by thick black lines, whereas the boundaries of data-driven regions are illustrated by thinner red lines. The thick black lines that are overlayed by thin red lines illustrate the matching boundaries of designated and data-driven regions. On the other hand, thin black lines illustrate the province boundaries, and each hub city is labelled by its name within the provincial boundary. We compared the overlap between the two regionalizations by comparing the percentage of shared and mismatched boundaries. We excluded the bordering boundaries to the surrounding seas and other countries. Overall, 22% of the MoH region boundaries did not match the boundaries of the data-driven regions of patient mobility. Eastern and south eastern regions of Diyarbakir, Elazig, and Van; Marmara regions of Tekirdag and Bursa; and inner Anatolian regions such as Kayseri and Konya perfectly matched with the designated regions of MoH. Some of the MoH designated regions consists of only one province, which is a policy of MoH to establish those provinces as hubs that could serve the surrounding regions. However, these provinces were merged with existing hubs and regions that are nearby in the data-driven regionalization. For example, Sanliurfa and Mersin, the two planned hubs, are merged with the existing hubs, Gaziantep and Adana, respectively. Also, Eskisehir province was considered as a major hub for the surrounding provinces by MoH. However, neighboring provinces of Eskisehir have stronger connections with other hubs such as Antalya and Izmir. The mismatch between the data-driven regionalization and designated regions by MoH can be used to implement policies that could strengthen the connection of provinces to their designated or planned regional hubs.

### Flow patterns

In this section we report the results and discuss the patterns we captured using the integrated visual analytics framework presented in “Multivariate clustering” section. We first selected the variables defined by the red subspace in Fig. 2 and incorporated these variables into the flow mapping and multivariate clustering framework to capture the monthly variation of patient flows across the 4-year period (Fig. 3). Green clusters of patients who are residents of eastern regions and seek health services in large cities of Istanbul, Ankara, Izmir and Adana from November to April. Patients from the north and north east regions represent the largest portion of these patterns and they primarily target Istanbul and north western regions, whereas patients from the south east target Adana. Patient movements are tightly related to the general mobility patterns in Turkey, which is driven by sociodemographic structure of the society and its history of urbanization. Istanbul has the ability to pull patients from greater distances as a function of its large population, health care infrastructure, as well as other motivations such as familiarity in the form of cultural and family ties with the rest of the country, and perceived quality of care [3]. Residents of Istanbul who originally migrated from distant areas tend to keep their ties to where they migrated from, often own second homes, and have relatives in those provinces. Patients that seek health care in these large cities often stay with relatives or connections through home town organizations [32, 33] when they receive their specialized medical care [32, 34–36]. Ankara and Izmir, the second and the third largest city in Turkey, also attract migrants however, not from far locations, rather from the nearby provinces. In contrast to the green clusters of flows that seek health services in big cities during winter months, purple clusters of patients in Fig. 3 travel to acquire health services during months between May and October. The purple clusters of patients are residents of large cities that seek care in the north, north-eastern and south-eastern regions. During summer months when the school is over, these migrants visit their relatives and use health care facilities in their hometowns in the east and northeast provinces. In addition, blue clusters of patient flows in Fig. 3 characterizes the short-distance mobility of patients mostly in the South-East. Patients in the South-East continue to use the services in the regional hub of Diyarbakir throughout the year, while the demand peaks during spring and autumn months.

**Fig. 9:**
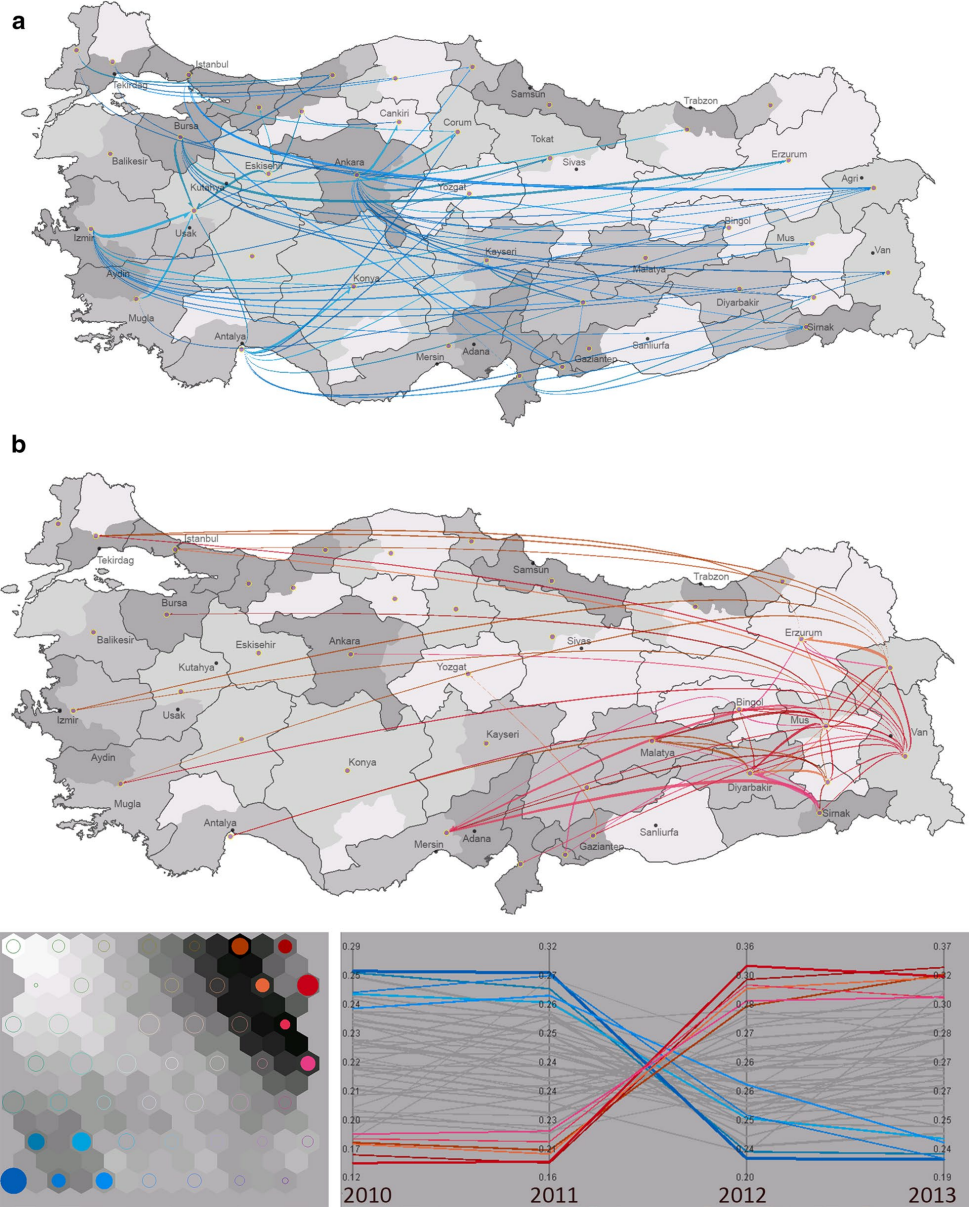
Patients seeking care from **a** central and western regions, **b** the south-east

Second, we visualize and explore the green subspace defined in Fig. 2 into the flow mapping and clustering framework in order to discover annual changes in mobility patterns. Using the visual analytics interface explained in Fig. 3, we select the flows that were high in all years to identify whether these flows had distinct changes in time and geographic space (Fig. 9). While the selection leaves the high volume of flows on the maps with red and blue clusters, the rest of the flows are hidden in the map view in order to highlight the details-on-demand (i.e., high values in all years) as part of the visual information seeking mantra [37]. Correspondingly, the values of flows that were filtered out are still displayed on the parallel coordinate plot with grey color to show the overall distribution while highlighting the blue and red clusters with dynamic brushing and linking. When Fig. 9a illustrates the blue clusters of patients that are residents of large cities who seek for health services in the eastern as well as less populated regions. These flows significantly declined after 2011. On the other hand, Fig. 9b illustrates the red clusters of patients are residents of south-eastern, eastern and southern regions that seek care in either the nearby hubs in the south and south-east or the north-west. These flows increased after 2011. We attribute the change in these mobility patterns after 2011 to the health care policy change implemented by the Turkish Health Transformation Program in 2011. The program was aimed to improve preventative care through family physicians, and thus, decrease the number of unnecessary admissions to secondary and tertiary care institutions. As a result, citizens were assigned to a family physician, and a considerable number of physicians in different specialties were assigned to the disadvantaged provinces in this period [28]. Our findings indicate that the program resulted in different outcomes across the country. The mobility of patients in the underdeveloped regions of the east and south east increased (Fig. 9b), while patient flows from the large cities to the eastern, south-eastern and other less populated areas across the country significantly decreased (Fig. 9a). The increasing mobility in the east and south east can be associated with the establishment of family doctors after 2011, which helped guide patients to seek for specialized care services in health care hubs.

**Fig. 10:**
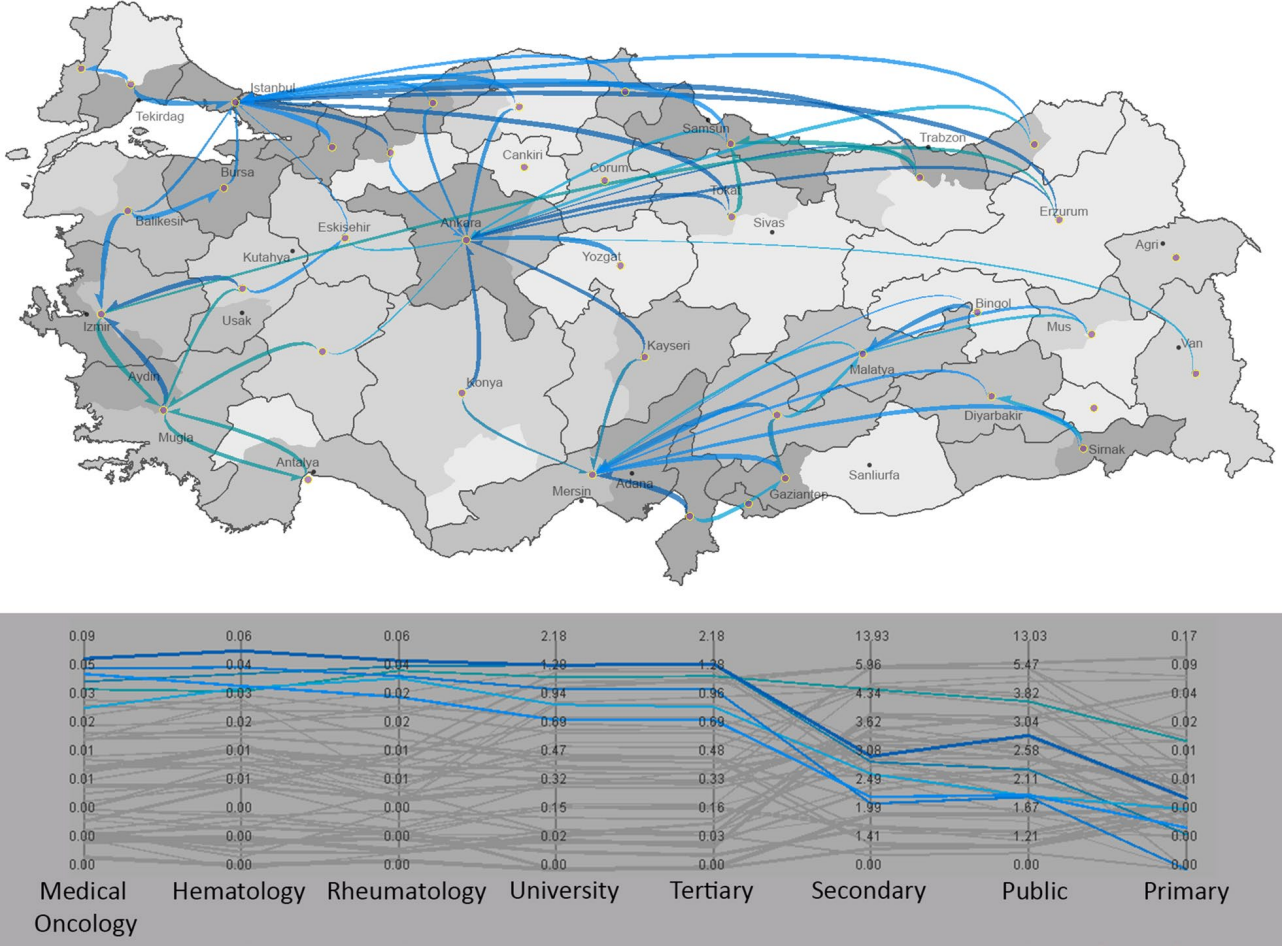
High proportion of patients seeking care in specialties such as medical oncology, hematology and rheumatology. Primary care demand for these patients were low, whereas secondary care and public institutions were utilized at medium level as compared to high level tertiary care and utilization of university hospitals

Third, we explore the yellow subspace in Fig. 2 which consists of flow variables by institution and medical service (Fig. 10). Within this subspace of variables, the most distinct pattern is characterized by the blue clusters of patients who seek specialized services in Medical Oncology, Hematology and Rheumatology at university hospitals and at the tertiary level of care. Primary care demand for these patients were low, whereas secondary care and public institutions were utilized at medium level as compared to high level tertiary care and utilization of university hospitals. Unsurprisingly, these flows were targeted at urbanized and developed regions of Ankara, Istanbul, Izmir, and Adana where there are university hospitals and tertiary care institutions that provide sufficient services for those specialized care demands.

## Discussion

We first discuss the implications of this study for the assessment of the health systems. Second, we discuss the limitations, challenges and future directions in the analysis of patient mobility. Our results agree with the findings of the previous work that analyze patient mobility data from European countries [5, 7, 38] in terms of the typology of patterns we discovered in our analysis. First, patient mobility correlates with provincial migration in the country. The provinces in western and eastern Marmara, Aegean, western and southern Anatolian regions have a relatively higher per capita income which attract migrants from the north-eastern Anatolian and mid-eastern Anatolian regions that are characterized by lower per capita income [20, 39]. The effect of social and geographical ties among various parts of the country is reflected in the use of health system. Patient movements to the large cities of Istanbul, Ankara and Izmir for receiving specialized medical care correlate with the migration patterns from the inner, north eastern and eastern parts of Anatolia, where the health infrastructure is inadequate in providing a comprehensive range of health services for the regional population. As a result of the lack of public policies and institutional support, patients from eastern Anatolia rely on family and kinship relationships to address their health care needs [40]. Second, different from the distant migratory ties, we identified a cluster of movements within the west, and south and south eastern regions, which may reflect strong cultural links. Patients living within these regions are more likely to look for treatment in areas that are culturally similar [3], and geographically close by for keeping the travel and health care related costs as minimum as possible.

In addition to socio-demographic characteristics that impact patient mobility, our results correlate with the policies implemented by MoH. After the centralized health care system patients were given the ability to go to private or government (public) health care institutions, and as a result, private institutions in the regions that lack health care infrastructure received large number of patients from surrounding regions. We also found a distinct shift in the mobility patterns after 2011 which we attribute to the health care policy change implemented by the Turkish Health Transformation Program in 2011 [41]. This policy assigned each citizen to a family physician. Our findings indicate that the program resulted in different outcomes across the country. As a result of guidance from family physicians, seeking for specialized medical care expanded in the underdeveloped regions of the east after the implementation of the family physician system. On the contrary, patient flows from the large cities to the eastern and south-eastern regions significantly decreased.

A major limitation of this study is that we were able to identify when, and where patient travel to receive what type of care, but we do not know the actual motivation whether it is affordability, perceived quality, or familiarity. Further studies, in the form of interviews could be beneficial to identify the motives behind patients’ choices. While our approach help identify flow patterns and trends, identifying main drivers of mobility requires comparing flow patterns to locational (node or regional) attributes such as socio-demographic and population characteristics and their spatiotemporal patterns. Patient mobility could result from a diverse set of reasons such as the number of hospitals/specialists, the number of beds, advanced health technology, lack of specialized centers, mistrust, comfort and cleaning of health care centers price, accessibility, seasonal migration and distances between origin and destination provinces [42–45]. Another factor is the presence of contact people through kinship and family ties at the destination as a result of past migration [36].

Because of high dimensionality (large number of attributes), and temporally varying characteristics, it is challenging to identify interesting relationships among the large number of flow and node attributes. There is a critical need for integrating feature selection methods to identify interesting relationships among a large number of both temporally varying variables such as health service capacity, population and patient characteristics. Also, in order to increase the quality of regionalization, and flow patterns, there is a need to increase the resolution of the dataset from province-to-province to district-to-district patient flows. While we hypothesize that the regional and national patterns would not change significantly, increasing the resolution of the dataset will allow capturing local patterns and fine details of regionalization.

## Conclusion

Our data-driven approach has two major contributions. First, we can identify medical regions from the patient mobility network, and compare them to the designated or planned regional structure of the health care system. The mismatch between medical regions of patient mobility and the designated regions highlight the fact that the patient use of health care services do not overlap with the planned structure of health service delivery by Ministry of Health (MoH). By pointing out malfunctioning medical regions and underutilized regional hubs, our study can directly be used in policy-making to improve the regional policy for health service delivery. Second, our study allows the identification of the demands, characteristics, and temporally varying patterns and shifts in patient mobility and health care utilization. Using such information, policies could be designed to satisfy varying needs of different regions such as the size, characteristics and health care demands of the population, and availability, quantity, quality and variety of health care providers. Implementing specific policies for different regions will allow addressing issues related to certain areas, and help close the gap between supply and demand for health service delivery.
